# Assessment of a Structurally Modified Alternanthera Mosaic Plant Virus as a Delivery System for Sarcoma Cells

**DOI:** 10.3390/v16101621

**Published:** 2024-10-16

**Authors:** Daria Fayzullina, Tatiana Manukhova, Ekaterina Evtushenko, Sergey Tsibulnikov, Kirill Kirgizov, Ilya Ulasov, Nikolai Nikitin, Olga Karpova

**Affiliations:** 1 Group of Experimental Biotherapy and Diagnostics, Institute for Regenerative Medicine, I.M. Sechenov First Moscow State Medical University, 119991 Moscow, Russia; dfaizullina@yandex.ru (D.F.); ser-tsibulnikov@yandex.ru (S.T.); ulasov75@yahoo.com (I.U.); 2 Department of Virology, Faculty of Biology, Lomonosov Moscow State University, 119991 Moscow, Russia; tanyafedorova0411@gmail.com (T.M.); nikitin@mail.bio.msu.ru (N.N.); 3 Research Institute of Pediatric Oncology and Hematology, N.N. Blokhin National Medical Research Center of Oncology, Ministry of Health of Russia, 115478 Moscow, Russia; kirgiz-off@yandex.ru

**Keywords:** plant virus, Alternanthera mosaic virus, Tobacco mosaic virus, structurally modified spherical particles, Ewing sarcoma, anticancer agents

## Abstract

The virions of plant viruses and their structurally modified particles (SP) represent valuable platforms for recombinant vaccine epitopes and antitumor agents. The possibility of modifying their surface with biological compounds makes them a tool for developing medical biotechnology applications. Here, we applied a new type of SP derived from virions and virus-like particles (VLP) of Alternanthera mosaic virus (AltMV) and well-studied SP from Tobacco mosaic virus (TMV). We have tested the ability of SP from AltMV (AltMV SP_V_) and TMV virions also as AltMV VLP to bind to and penetrate Ewing sarcoma cells. The adsorption properties of AltMV SP_V_ and TMV SP are greater than those of the SP from AltMV VLP. Compared to normal cells, AltMV SP_V_ adsorbed more effectively on patient-derived sarcoma cells, whereas TMV SP were more effective on the established sarcoma cells. The AltMV SP_V_ and TMV SP were captured by all sarcoma cell lines. In the established Ewing sarcoma cell line, the effectiveness of AltMV SP_V_ penetration was greater than that of TMV SP. The usage of structurally modified plant virus particles as a platform for drugs and delivery systems has significant potential in the development of anticancer agents.

## 1. Introduction

Plant viruses, both in the form of virions or virus-like particles (VLP) and structurally modified particles (SP), are of significant interest in biotechnology and biomedicine, particularly for vaccine development and cancer treatment.

Plant viruses are non-pathogenic for mammals and safe for human use, and no serious adverse events have been reported [1]. Large quantities of these viruses readily accumulate in plants, and the extraction and purification of plant viruses are effective (and economically feasible in terms of time and materials). Moreover, many plant viruses remain stable, in particular under physiological conditions. The immune system recognizes such particles well and induces both cell and humoral response [2,3,4,5].

A considerable amount of accumulated data show that SP, VLP, and virions produce an adjuvant effect in relation to a wide range of antigens that differ in nature and size. Moreover, promising data have been obtained on the application of the virion and virus-like particles of plant viruses as anticancer agents [6,7]. They can act as adjuvants in cancer immunotherapy and can be used as drug delivery systems for cancer cells. For instance, it was shown that SP from Tobacco mosaic virus (TMV, genus *Tobamovirus*, family *Virgaviridae*) induce antitumor immunity, leading to a slowdown in melanoma growth [8]. Moreover, TMV SP conjugated with doxorubicin or containing it inside the particles have an inhibitory effect on breast cancer cells [9]. However, investigations of the potential use of SP in anticancer treatment should not be restricted to TMV SP alone. Other plant viruses, in particular representatives of families *Alphaflexiviridae* (Potato virus X), *Tymoviridae* (Physalis mottle virus) and *Secoviridae* (Cowpea mosaic virus), can also be used to obtain new biogenic platforms [10,11,12,13,14,15,16,17].

Here, for the first time, we employed a new type of SP from Alternanthera mosaic virus (AltMV) to study the possibility of targeting cancer cells. The Alternanthera mosaic virus (the strain AltMV-MU (Moscow University) FJ822136) is a member of the genus *Potexvirus*, family *Alphaflexiviridae*. In contrast to rod-like TMV, the AltMV virions are helical filamentous particles. The AltMV virion consists of RNA and coat protein (CP). The AltMV CP self-assembles in vitro in the absence of viral RNA into virus-like particles (VLP) [18]. It was previously shown that SP obtained from AltMV virions (SP_V_) and AltMV VLP (SP_VLP_) expose lysine and cysteine amino acids on their surface [19]. Notably, in comparison with TMV SP, AltMV SP have a greater perspective for target molecule conjugation, including with chemotherapeutic agents, due to the greater number of reactive amino acid residues in the coat protein.

Considering the possibility of using AltMV SP for chemotherapeutic loading in the future, we first focused on studying the AltMV SP adsorption and penetration into sarcoma cells. Ewing sarcoma (ES) is an aggressive tumor of mesenchymal origin that accounts for 10% to 15% of all bone sarcomas, according to various data and classifications [20]. Over the past 40 years, there have been significant advances in the treatment of localized tumors, with 5-year survival rates increasing from 20% to just over 70%. However, ES is diagnosed in the presence of metastases in one-quarter of cases, and the relapse rate remains high (25%). In this group, the 5-year survival rate for patients with a disease-free interval (DFI) > 2 years is approximately 30%, and the 5-year survival rate for those with a DFI of 2 years is approximately 7%. In addition, there is no standard therapy for recurrent ES [21]. This disease requires the development of new therapeutic approaches and improvements in old patients. The use of a delivery system may increase the effectiveness of ES therapy while reducing the incidence of side effects. The present work is dedicated to examining the adsorption and penetration abilities of AltMV SP and TMV SP as delivery systems for sarcoma cells.

## 2. Materials and Methods

### 2.1. Virus Purification and Coat Protein Isolation

TMV and AltMV were isolated and purified as previously described [19,22]. The AltMV coat protein was isolated via salt deproteinization with 2 M LiCl, as described previously [18].

### 2.2. Formation of Structurally Modified Particles and Their Labeling with Fluorescein Isothiocyanate

SP from TMV at a concentration of 0.1 mg/mL were obtained according to [22]. SP from AltMV virions and VLP at a concentration of 1 mg/mL were generated as previously described [19]. All types of SP were labeled with fluorescein isothiocyanate (FITC) (Sigma-Aldrich, St. Louis, MO, USA, 3326-32-7) according to the manufacturer’s protocol, with some modifications described in [19]. Before labeling, TMV SP were concentrated by refrigerated CentriVap centrifugal concentrator (Labconco, Kansas City, MO, USA). The labeling results were visualized via the Axiovert 200 M fluorescence microscope (Carl Zeiss, Gottingen, Germany) equipped with a digital cooled camera ORCAII-ERG2 (Hamamatsu Photonics K.K, Hamamatsu City, Japan) and by SDS-PAGE (8–20%) electrophoresis with subsequent analysis under UV light via the ChemiDoc^TM^; XRS+ System with Image Lab^TM^ Software (Bio-Rad Laboratories, Hercules, CA, USA).

### 2.3. Transmission Electron Microscopy

The samples were contrasted by 2% uranyl acetate and examined by transmission electron microscopes JEM-1400 (JEOL, Tokyo, Japan) and JEM-1011 (JEOL, Tokyo, Japan). The image processing software designed for scientific multidimensional images, ImageJ version 1.52a (National Institutes of Health, Stapleton, NY, USA) was used for diameter calculations.

### 2.4. Nanoparticle Tracking Analysis

The experiments were performed with NanoSight NS500 instrument (NanoSight, Salisbury, UK) equipped with a 532 nm green laser and a high-sensitivity EMCCD camera Andor Luca. The samples were diluted with Milli-Q to an approximate total particle concentration of 1 × 10^8^–3 × 10^8^ particles/mL. At least 20 individual measurements for 30 (TMV SP and AltMV SP_VLP_) or 60 s (AltMV SP_V_) were collected for each sample. The videos were processed with NTA 2.3 software version 2.3 build 0033 (NanoSight, Salisbury, UK). At least 1800 tracks for individually tracked particles were collected for each SP sample. The hydrodynamic diameter (nm) and total particle concentration (particles/mL) were determined.

### 2.5. Cells and Reagents

The patient-derived T46 and ES36 Ewing sarcoma cells were created following a previously published procedure [23]. The created primary cell cultures were evaluated by immunofluorescence (to detect CD99 and SOX2 cellular markers) and PCR (to detect EWS/FLI1 fusion). Multiplex PCR of 22 loci with STR repeats (SBT-RealGene SCREEN, SystemBiotech, Moscow, Russia) was used to authenticate the cell cultures. Dr. Sergey Boychuk (Kazan Federal University, Kazan, Russia) provided A673 Ewing sarcoma cells. All sarcoma cells were grown in RPMI-1640 medium (Paneco, Moscow, Russia) supplemented with 10% fetal bovine serum (Intl Kang, Global Kang Biotechnology, Beijing, China) and 1% penicillin–streptomycin (Kino Co., Ltd., Hangzhou, China) in humidified atmosphere with 5% CO_2_/95% air at 37 °C. As a control, the normal fibroblast cell line M19 [24] was employed.

### 2.6. Adsorption of SP on Cells

Adsorption was performed in a 48-well plate. The cells were seeded into the plate within 24 h (RPMI or DMEM supplemented with 10% FBS str/pen, working volume 500 μL, and cell concentration 20,000 cells per well). The concentration of AltMV SP_V_, AltMV SP_VLP_, and TMV SP was 1.3 × 10^9^ particles per well in serum-free medium. Cells were incubated with particles for 1 h at +4 °C in the dark in a sterile laminar chamber according to a standard protocol for the adsorption of viral particles on cells [25]. The aforementioned conditions preclude the cells from endocytosis, thereby enabling the outcome of adsorption to be observed independently of other types of interactions, such as penetration, between cells and particles [13]. The medium containing the SP was then removed and the cells were washed with sterile PBS. As negative control were used cells without the addition of SP. Adsorption results were recorded using a green laser (λ = 488 nm) on an EVOS M5000 fluorescence microscope (ThermoFisher, Waltham, MA, USA). For each well, 5 fields were recorded in two channels: light microscopy and fluorescence microscopy with a green laser. Overlaid channels were analyzed using the ImageJ (software version 1.53e, National Institutes of Health, Stapleton, NY, USA) software to count the total number of cells (*n_i_*) and the number of cells, which adsorbed FITC-labeled SP (*x_i_*). Adsorption effectiveness was calculated as *x_i_/n_i_* for each field. The data were reported as the mean effectiveness ± standard deviation across all 5 fields.

To compare the overall adsorption effectiveness of different SP across all the studied tumor cells, the adsorption effectiveness ratio (AER) was used. For each SP, *n* and *x* were pooled over all sarcoma cells, followed by calculation of the ratio with indices 1 and 2 corresponding to different SP:

AER=x1n1x2n2

From a mathematical point of view, *x_i_* positive adsorption events out of *n_i_* observations represent the sample from the binomial distribution with adsorption probability *p_i_*. For the ratio of probabilities from two samples of this kind, an efficient and robust estimator of the confidence interval was proposed by [26]. The natural logarithm for the 95% CI of the AER could be calculated as follows:

$$Ln\left(AER\right)\pm 1.96\;\sqrt{\frac{n_{1}-x_{1}}{n_{1}\;x_{1}}+\frac{n_{2}-x_{2}}{n_{2}\;x_{2}}}$$

The 95% CI was calculated by exponentiation of these bounds.

### 2.7. Cellular Uptake

Within 24 h, the cells were seeded in a 24-well plate (RPMI or DMEM supplemented with 10% FBS str/pen, working volume 500 μL, cell concentration 40,000 cells per well). The concentrations of AltMV SP_V_, AltMV SP_VLP_, and TMV SP were 1.3 × 10^9^ particles per well in serum-free medium. Cells with SP were incubated in a CO_2_ incubator at 37 °C for 5 h according to [9] with a modified particle-to-cell ratio and incubation time. After incubation, the medium containing SP was removed and the cells were washed with sterile PBS. Cells were harvested, centrifuged, and resuspended in the flow cytometry buffer (PBS). It is important that despite the washes, some of the SP remained adsorbed on the cells surface. Thus, the cumulative effect of penetration and adsorption was registered. The results were recorded using a green laser (λ = 488 nm) on a flow cytometer (Sony SH800, Sony Biotechnology, Tokyo, Japan). As negative control were used cells without the addition of SP. Analysis of each sample was stopped when 10,000 events were recorded.

### 2.8. Immunofluorescence Assessment of AltMV SP_V_ Localization inside Target Cells

ES36 were grown on coverslips (2 × 10^5^ cells per coverslip) covered with 1% laminin 24 h prior to treatment. The next day, the cells were washed and FITC-conjugated AltMV SP_V_ (1.3 × 10^9^ particles per well) were added to the well and incubated for 5 h at 37 °C in humidified CO_2_. After 12 h, the cells were fixed with 4% methanol, blocked with 1% FBS, and stained with rhodamine–phalloidin-labeled antibodies against F-actin (Thermo Fisher Scientific, Waltham, MA, USA) following by counterstaining with mounting solution enriched with Hoechst 33342 (NucBlue^TM^ Live ReadyProbes^TM^, Thermo Fisher, Waltham, MA, USA). Images were acquired using Olympus Fluoview FV3000 (Olympus Corporation, Tokyo, Japan, software FV31S-SW), confocal microscopy, and analyzed using Image J (software version 1.53e, National Institutes of Health, Stapleton, NY, USA) software.

### 2.9. Statistical Analysis

Graphing and statistical analysis were performed using Prism GraphPad software 9.5.1 (GrahPad Software Inc., San Diego, CA, USA). The results of the SP adsorption were analyzed by an ordinary one-way ANOVA with Dunnett’s post hoc test. For quantitative analysis of flow cytometry data and comparison of TMV SP and AltMV SP_V_ groups, Welch’s *t*-test was used. *p*-values of less than 0.05 were considered to be significant.

## 3. Results

### 3.1. Characterization of SP

**Figure 1:**
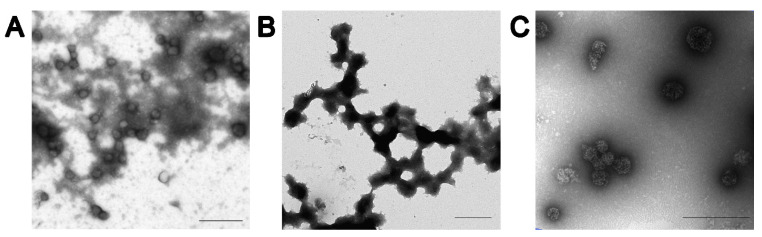
Morphology of spherical particles obtained from (**A**) Tobacco mosaic virus virions, (**B**) Alternanthera mosaic virus (AltMV) virions, and (**C**) AltMV virus-like particles. Scale bar 500 nm. Transmission electron microscopy.

Three types of FITC-labeled SP were used for this study: TMV SP, AltMV SP_V_, and AltMV SP_VLP_. The mean hydrodynamic diameters determined by the nanoparticle tracking analysis were in the range of 107–114, 182–186, and 113–124 nm for TMV SP, AltMV SP_V_, and AltMV SP_VLP_, respectively. It has been confirmed by transmission electron microscopy that all particles have morphologies close to spherical (Figure 1). Fluorescence microscopy and electrophoretic analysis in 8–20% SDS-PAGE followed by UV light detection confirmed that all particles were successfully labeled with FITC (Figures S1 and S2). The qualitative analysis has shown that AltMV SP_VLP_ (Figure S2, lanes 1–3) and AltMV SP_V_ (Figure S2, lanes 4–6) were labeled more effectively than TMV SP (Figure S2, lanes 7–9).

### 3.2. Experimental Design and SP Adsorption

**Figure 2:**
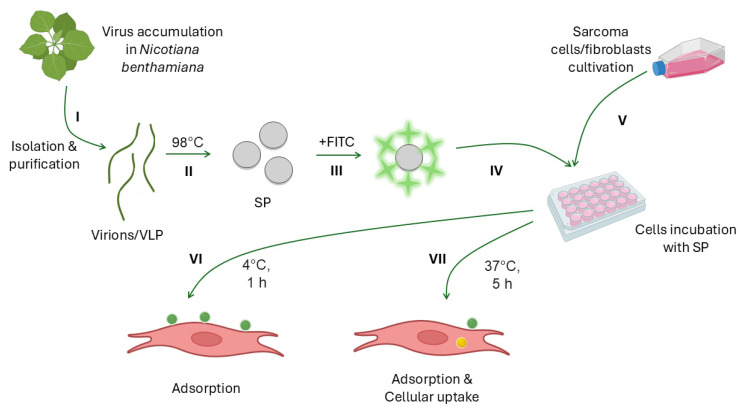
Scheme of the experiment. (**I**) Virus isolation and purification; obtaining of virus-like particles (VLP); (**II**) formation of spherical particles (SP) after the heating of virions and VLP; (**III**) SP labeling with fluorescein isothiocyanate (FITC); (**IV**) plating of cells 24 h prior to treatment with SP; (**V**) incubation of cells with SP; (**VI**) adsorption of SP on the cellular membrane of sarcoma and control cells (fibroblasts); and (**VII**) the cumulative effect of adsorption (green) and uptake (yellow). Three types of SP were used in the experiments: SP from Tobacco mosaic virus, SP from Alternanthera mosaic virus (AltMV), SP from AltMV VLP.

The FITC-labeled SP were used in the experiments to study their potential as a delivery system. Their ability to adsorb onto the surface of sarcoma cells and subsequently penetrate them was examined. The experimental procedure was divided into the seven steps presented in Figure 2.

**Figure 3:**
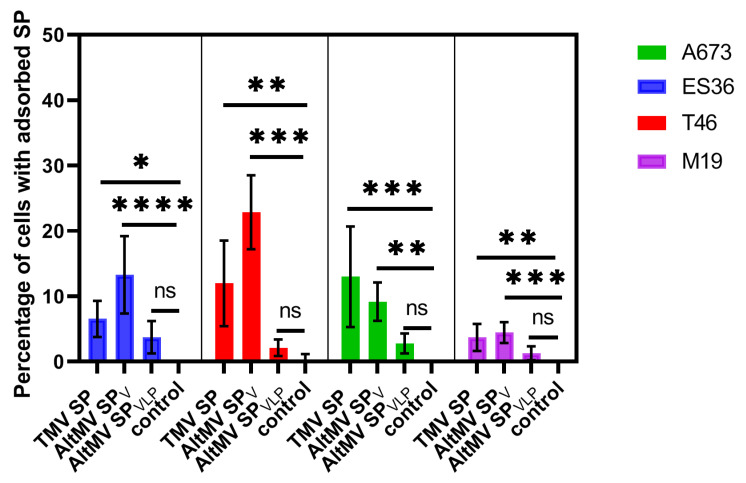
Adsorption effectiveness of fluorescein isothiocyanate-labeled spherical particles (SP) on T46, ES36, M19, and A673 cells. The data are presented as the means and standard deviations of five technical repeats. The ordinary one-way ANOVA with Dunnett’s post hoc test was used for multiple comparisons of the adsorption of each SP type with that of the control for each cell line. * *p* < 0.05, ** *p* < 0.01, *** *p* < 0.001, **** *p* < 0.0001, ns—not significant. ES36, T46—primary patient-derived Ewing sarcoma cells, A673—established Ewing sarcoma line, M19—normal fibroblast cell line. Control—cells without the addition of SP. TMV SP—SP obtained from tobacco mosaic virus, AltMV SP_V_—SP obtained from alternanthera mosaic virus (AltMV) virions, AltMV SP_VLP_—SP obtained from AltMV virus-like particles.

The experiments were performed on two Ewing sarcoma patient-derived short-lived cell lines (T46 and ES36), the established sarcoma line A673, and fibroblasts (cell line M19). At first, we studied the difference in the effectiveness of the adsorption of TMV SP, AltMV SP_V_, and AltMV SP_VLP_ on three tumor lines and healthy cells. The percentage of cells with adsorbed SP was used to compare the adsorption effectiveness of different particles and their effectiveness across different cell lines. When comparing the adsorption effectiveness of different SP within a single cell line, both TMV SP and AltMV SP_V_ adsorbed on the surface of all cell lines (Figure 3). However, although AltMV SP_VLP_ adsorbed on sarcoma cells better than the control, this difference was not statistically significant.

Next, the adsorption effectiveness of SP on all sarcoma cell lines, regardless of their primarity or constitutivity, was analyzed. For this purpose, pairwise comparisons of adsorption effectiveness (relative adsorption or adsorption efficiency ratio (AER)) were performed. It was demonstrated that, for all tumor cell lines studied, AltMV SP_V_ were adsorbed 4.7 times more effectively than AltMV SP_VLP_ (95% CI from 3.5 to 6.3). Similarly, TMV SP were adsorbed 3.9 times more effectively than AltMV SP_VLP_ (95% CI from 2.9 to 5.2). Thus, the adsorption effectiveness of AltMV SP_V_ and TMV SP on sarcoma cells was comparable. Since the confidence intervals do not include one, both effects are statistically significant. This verifies that AltMV SP_V_ adsorb on sarcoma cells at least comparably to TMV SP, whereas AltMV SP_VLP_ adsorb on sarcoma cells much less effectively.

**Figure 4:**
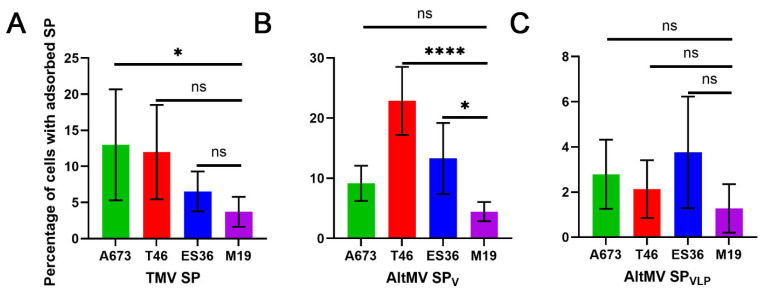
Comparison of the adsorption of different fluorescein isothiocyanate-labeled spherical particles (SP) by sarcoma cell lines and fibroblasts (M19). The data are presented as the mean of five technical repeats and standard deviations. The ordinary one-way ANOVA with Dunnett’s post hoc test was used for multiple comparisons of the adsorption of each SP type with that of the control for each cell line. * *p* < 0.05, **** *p* < 0.0001, ns—not significant. ES36, T46—primary patient-derived Ewing sarcoma cells, A673—established Ewing sarcoma line, M19—normal fibroblast cell line. Control—cells without the addition of SP. The adsorption of SP obtained from (**A**) Tobacco mosaic virus virions (TMV SP), (**B**) Alternanthera mosaic virus virions (AltMV SP_V_), and (**C**) AltMV virus-like particles (AltMV SP_VLP_) on sarcoma cell lines and fibroblasts (M19).

Then, we analyzed the same data to assess how well each type of SP adsorbs on three sarcoma lines compared to fibroblasts. It was found that TMV SP adsorbed more effectively on the established sarcoma cell line A673 than on fibroblasts. However, statistically significant adsorption was not demonstrated on primary sarcoma lines (Figure 4A). In contrast, AltMV SP_V_ adsorbed more effectively on the primary cell lines T46 and E36 compared to M19, while no difference was observed on A673 (Figure 4B). Nevertheless, similar to the previous analysis (Figure 3), AltMV SP_VLP_ did not demonstrate any better adsorption ability on sarcoma cells than on fibroblasts (Figure 4C).

Given that primary sarcoma cell lines are more closely related to native sarcoma cells, we suggest that AltMV SP_V_ show better binding efficacy on these cell lines (Figure 4B), which makes them a more promising delivery system. Therefore, the results obtained on T46 and ES36 are more likely to reflect the expected outcomes in situ.

Apart from that, another important aspect of adsorption is the specificity and selectivity of a delivery system. The high binding efficiency of AltMV SP_V_ specifically on sarcoma cells compared to fibroblasts suggests that these particles are more selective for the sarcoma cell surface. Considering also the possibilities of further modifications of SP, the natural specificity of AltMV SP_V_ for tumor cells characterizes these particles as a delivery system with high potential in Ewing sarcoma (ES) therapy.

Hence, the presented data show that both TMV SP and AltMV SP_V_ adsorb on sarcoma cells at similar levels, while AltMV SP_VLP_ did not demonstrate effectiveness in the comparisons conducted. Consequently, AltMV SP_VLP_ were not used for the subsequent experiments.

### 3.3. Cellular Uptake and Retention of SP by Primary Tumor Cells

Next, we assessed the ability of SP to penetrate cells, as an effective drug delivery system requires effective uptake by target cells. FITC-labeled SP were incubated at 37 °C with ES36, T46, A673, and M19 cells. It should be emphasized that under the experimental conditions, both SP uptake and adsorption may occur. Therefore, the obtained results reflect the cumulative effect of these two processes (Figure 2, step VII).

**Figure 5:**
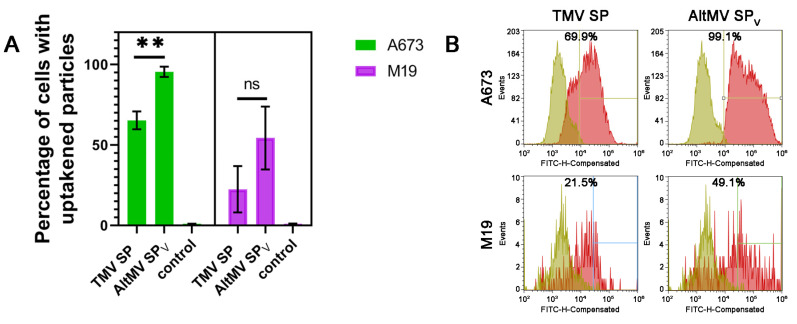
Spherical particles (SP) cellular uptake. (**A**) Graphical representation of the cellular uptake results according to the Sony SH800 flow cytometer for SP in A673 and M19 cells. For quantitative analysis of the efficiency of SP penetration into cells, the experiment was performed in triplicates. Statistical significance was analyzed using Welch’s *t*-test, ** *p* < 0.01, ns—not significant. (**B**) Distribution of cells incubated with SP according to fluorescence intensity. The events recorded by the device are plotted along the ordinate axis. The abscissa axis shows the fluorescence intensity detected in the channel. Control—cells without the addition of SP. The control is imposed on the results obtained during incubation with particles. It determines the boundary (vertical line), after which the result is considered positive. TMV SP—SP obtained from Tobacco mosaic virus, AltMV SP_V_—SP obtained from Alternanthera mosaic virus virions.

First, we conducted a qualitative assessment of the penetration ability of AltMV SP_V_ and TMV SP on the primary and established sarcoma cell lines compared to fibroblasts. According to Table 1, both types of particles are captured by both tumor and healthy cells (Figure S3). To determine if there are any quantity differences in penetration ability between AltMV SP_V_ and TMV SP, several experimental repeats were performed on fibroblast and the established cell line A673. The reason we chose A673 cells is that they will be utilized for the in vivo binding experiments of AltMV SP_V_ and TMV SP as well as the delivery of therapeutic payloads. It was shown that AltMV SP_V_ penetrate tumor cells more effectively than TMV SP (Figure 5A). Histograms of cell distribution by fluorescence intensity after the capture of TMV SP and AltMV SP_V_ (Figure 5B and Figure S4) also confirm the higher effectiveness of AltMV SP_V_ penetration. The majority of A673 cells after incubation with AltMV SP_V_ were positioned further from the control sample on the chart compared to TMV SP. However, this distribution was not observed on the fibroblasts, indicating the specificity of AltMV SP_V_ for sarcoma cells.

**Figure 6:**
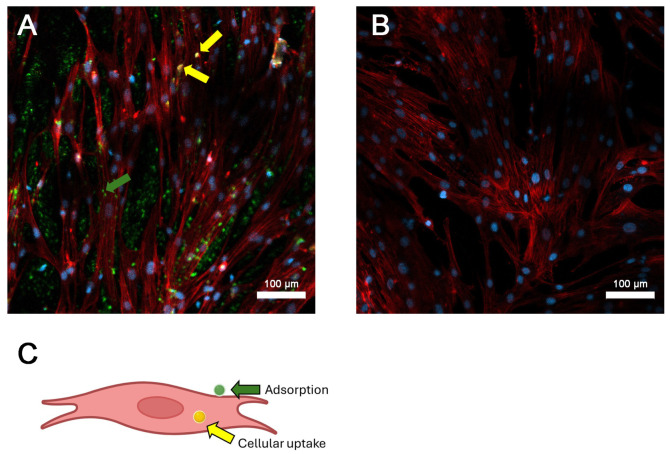
Retention of spherical particles (SP) obtained from Alternanthera mosaic virus virions (AltMV SP_V_) inside primary Ewing sarcoma cells (ES36). (**A**) AltMV SP_V_ adsorption and penetration by ES36 cells. (**B**) Negative control—ES36 without added AltMV SP_V_. (**C**) Scheme of the color marks distribution produced by the green signal of fluorescein isothiocyanate-labeled AltMV SP_V_ and the red signal of rhodamine–phalloidin-colored F-actin. Colocalization of the green and red signals produces a yellow signal (yellow arrows), indicating the penetration of SP into the cell. The green signal demonstrates AltMV SP_V_ that did not penetrate the cells and remained on the membrane and substrate surfaces (green arrow). For the confocal microscopy, the magnification was 20.

We also studied the distribution of penetrated FITC-labeled AltMV SP_V_ in ES36 Ewing sarcoma cells using confocal microscopy (Figure 6, 20× magnification; Figure S5, 40× magnification). To identify the localization of FITC-labeled AltMV SP_V_, we stained F-actin with rhodamine–phalloidin and nucleus with Hoechst 33342. A yellow signal, resulting from the overlay of rhodamine–phalloidin and FITC signals, confirms that AltMV SP_V_ accumulate in the cytoplasm of ES36 cells (Figure 6A, yellow arrows). Simultaneously, a green signal produced by FITC (Figure 6A, green arrows) indicates AltMV SP_V_ adsorbed on the cell membrane or laminin glass substrate. This corresponds to the combined effects of uptake and adsorption observed under the experimental conditions.

Therefore, here we demonstrated that AltMV SP_V_ penetrates both primary and established tumor cell lines, with the penetration ability of AltMV SP_V_ being higher on the established sarcoma line compared to TMV SP.

## 4. Discussion

While it is clear that oncology research must focus on developing novel medications, active molecules, and combination therapy approaches, an important component of care that is sometimes overlooked is the efficient delivery of therapeutic payloads to cancer cells [9]. Today, there are many different classes of materials, some of which are in development, and some of which have even completed all stages of testing and have been introduced into clinical practice. For example, Abraxane^®^, which is based on albumin, and Doxil^®®^, which is a liposomal drug, are being used in the clinic. Particular attention has recently been paid to the use of plant viruses in cancer treatment and the consequent creation of several tumor-targeting platforms for use in cancer treatment [9,27,28,29].

Plant viruses have several advantages over other natural and artificially synthesized materials. They are safe for humans and animals and are unable to be replicated in mammalian cells [1]. Compared with synthetic materials, plant virus particles are biocompatible, biodegradable, and easily cleared from laboratory animals, giving them an advantage over synthetic materials [1,30]. The rapid clearance of TMV particles from non-target organs and tissues has been demonstrated for TMV [3]. Plant viruses can be administered at doses up to 100 mg per kilogram of body weight without clinical toxicity [9,31]. In addition, virus particles are relatively easy and rapid to grow in plants, and their production is inexpensive and scalable [32]. Moreover, many plant viruses are highly susceptible to chemical, thermal, and genetic modifications to produce stable forms [33]. It should be mentioned that using plant virus-based platforms faces a problem of clearance by the immune system. However, there are several strategies allowing “camouflage” plant virus-based particles/virions and overcome this issue [34,35,36]. One of the most common approaches of particles shielding is PEGylation [3,16,36]. Coating plant viral particles with PEG may reduce immune response and blood clearance. However, there are studies demonstrating the presence of antibodies to PEG in up to 25% of the human population [37]. These findings raise a debate about the use of PEG in clinical practice. One of the possible causes of antibodies to PEG in the human population is the widespread application of PEG in the manufacturing of different products, including drugs and cosmetics. However, in addition to PEG, there are studies showing the promise of other agents that can hide viral particles from the immune system, for example, silica or serum albumin [34,35].

In agreement with the above benefits, several studies have described the use of plant viral-based particles for targeting cancer cells. For instance, TMV SP were explored for their ability to deliver doxorubicin to breast cancer cells [9]. This time, we have used AltMV SP to target Ewing sarcoma (ES) cells, thus expanding the number of SP for anticancer therapy. Adolescents are the main victims of ES, an aggressive mesenchymal cancer that necessitates changing current treatment plans because of the dismal 5-year survival rate of patients, especially those with metastases. From other sites, a high degree of ES heterogeneity, which benefits from genetic and epigenetic cellular reprogramming, displays poor differentiated cellular populations inside ES tumors with potential for tumor growth and invasion. Clinically valuable, therefore, is the use of patient-derived cellular cultures to solve the problem of drug delivery specificity to certain malignancies, such as Ewing sarcoma. Thus, testing for adhesion and penetration to the main tumor cells proved clinically beneficial.

A particle needs to possess three key characteristics to be used as a delivery system. The first is the ability to attach an active medication to the particles. Our group has previously shown that AltMV SP_V_ and AltMV SP_VLP_ contain lysine and cysteine residues that are potentially accessible for conjugation [19]. Compared with TMV SP, AltMV SP may be more promising for bioconjugation because, on average, the AltMV coat protein contains more amino acid residues that can be used for bioconjugation (eight lysine residues, two cysteine residues, and fourteen glutamic and aspartic acid residues) than the TMV coat protein. The results of this study confirm our assumption, since labeling AltMV SP with fluorescein isothiocyanate is qualitatively more effective than labeling TMV SP. Secondly, the particles must also have the ability to adhere to target cells. Although, undoubtedly, in the initial step towards the successful implementation of delivery—further system penetration inside—the reversibility or irreversibility of adsorption in this case is irrelevant. The best outcome for adsorption should also provide tumor cell specificity, but particle modifications are known to increase nanoparticle adherence [38]. Our results demonstrate the specificity of naked SP from AltMV virions for adherence to ES cells, and this effect can be later elevated by further making structural modifications to spherical particles. Specifically, for the first time, we show that the adsorption of TMV SP and AltMV SP_V_ on ES cells and AltMV SP_V_ exhibit better adsorption on primary ES cultures among AltMV SP_VLP_ and TMV SP. Further work with these SP on Ewing sarcoma was also stopped since, when comparing the adsorption level of AltMV SP_VLP_ particles to the negative control, we could not find a statistically significant difference. Maybe more research may be carried out to examine these particles’ potentials for use with different kinds of tumors. Although the potential primary receptor for the AltMV developed particles it remains unclear, as well as their distribution on the surface of healthy and tumor cells, the increased adherence of AltMV SP_V_ to the membrane of primary ES cells suggests an increased density of unknown primary moieties in the culture of primary ES cells. Of note, the culture of human embryonic fibroblasts M19 displays a low level of AltMV SP_V_ binding, which highlights the cellular specificity of AltMV SP_V_ as a drug delivery platform in oncology applications.

Finally, as seen earlier, the delivery system for a vehicle intended to target tumor cells needs to be able to penetrate the cell membrane and release a payload inside the target cells. We demonstrated that AltMV SP_V_ after binding to the membrane of target cells penetrates and retains within the cytoplasm. Although such an effect was seen in up to 5–8% of target cells, the tumor-penetrating ability can be increased by retargeting AltMV SP_V_ to an alternative receptor such as neuropeptide Y1 [39], IGF-1R [40], or unknown surface proteins detected in our earlier investigation [23]. Previously, the effectiveness of stimulating the antitumor response in B16F10 melanoma cells was demonstrated for TMV SP. TMV SP and TMV virions both slowed down the rate of tumor growth in mouse studies, extending the animals’ lives [8]. Using two cell lines of breast cancer, the same group of scientists showed that TMV SP could be conjugated with a chemotherapy agent such as doxorubicin, demonstrating both the drug’s effective release and the death of cancer cells. Furthermore, compared to TMV virions, the rate at which cells absorbed TMV SP was noticeably higher [9]. Although the colleagues did not continue their research with TMV SP, based on the data obtained in the work, we believe that the potential of using structurally modified particles as a platform for drug development and delivery is far from exhausted. The study of new structurally modified particles such as AltMV SP_V_ may allow for the design of new drug delivery systems to tumor cells.

## 5. Conclusions

In summary, we have carried out pilot research to examine the potential of using structurally modified plant viruses as a platform for anticancer therapy. Structurally modified plant viruses hold great potential and offer a number of unique advantages since plant viruses are not capable of infecting human cells and are biocompatible with low-cost production. Here, we have shown for the first time that AltMV SP_V_ as well as TMV SP can adsorb on and penetrate Ewing sarcoma cells, opening up the possibility of using them as antitumor drug delivery systems.

## Figures and Tables

**Table 1:** Qualitative analysis of the ability of spherical particles (SP) to penetrate healthy M19 cells, the established A673 cell line, and patient-derived short-lived Ewing sarcoma cultures ES36 and T46. TMV SP—SP obtained from Tobacco mosaic virus, AltMV SP_V_—SP obtained from Alternanthera mosaic virus virions.

Cell Line	Type of SP	SP Cellular Uptake
ES36	TMV SP	8.0%
	AltMV SP_V_	23.7%
T46	TMV SP	16.3%
	AltMV SP_V_	21.5%
A673	TMV SP	85.4%
	AltMV SP_V_	99.6%
M19	TMV SP	4.0%
	AltMV SP_V_	20.4%

## Data Availability

All the relevant data are provided in this paper and in the Supplementary Materials.

## Supplementary Materials

The following supporting information can be downloaded at: https://www.mdpi.com/article/10.3390/v16101621/s1, Figure S1: SP fluorescence labeling. A, B—TMV SP; C, D—AltMV SP_V_; E, F—AltMV SP_VLP_. A, C, E—phase contrast image; B, D, F—fluorescence image. Scale bar 5 μm; Figure S2: FITC-labeled coat protein within the following structures: lanes 1–3—AltMV SP_VLP_, lanes 4–6—AltMV SP_V_, lanes 7–9—TMV SP. Lanes 1, 2, 4, 5, 8, 9–1 µg, lanes 3, 6, 7–2 µg. A—staining with Coomassie G-250; B—visualization in UV-light; Figure S3: SP cellular uptake. Distribution of cells incubated with SP by fluorescence intensity for qualitative analysis. The events recorded by the device are plotted along the ordinate axis. The abscissa axis shows the fluorescence intensity detected in the channel. Control—cells without the addition of SP. The control is imposed on the results obtained during incubation with particles. It determines the boundary (vertical line), after which the result is considered positive. ES36, T46—primary patient-derived Ewing sarcoma cells, A673—established Ewing sarcoma line, M19—normal fibroblast cell line; Figure S4: SP cellular uptake. Distribution of cells incubated with SP by fluorescence intensity for qualitative analysis. The events recorded by the device are plotted along the ordinate axis. The abscissa axis shows the fluorescence intensity detected in the channel. Control—cells without the addition of SP. The control is imposed on the results obtained during incubation with particles. It determines the boundary (vertical line), after which the result is considered positive. A) Distribution for SP added to M19—normal fibroblast cell line. B) Distribution of SP added to the A673—established Ewing sarcoma line; Figure S5: AltMV SP_V_ retention inside primary Ewing sarcoma cells (ES36). A. AltMV SP_V_ adsorption and penetration by ES36 cells. B. Negative control—ES36 without added AltMV SP_V_. Colocalization of the green and red signals produces a yellow signal, indicating the penetration of SP into the cell. The green signal demonstrates AltMV SP_V_ that did not penetrate the cells and remained on the membrane and substrate surfaces. For the confocal microscopy, the magnification was 40.
