# Supplementary material for: Assessment of a Structurally Modified Alternanthera Mosaic Plant Virus as a Delivery System for Sarcoma Cells
Source: Viruses. 2024 Oct 16;16(10):1621. doi: 10.3390/v16101621 (PMC11512230; doi:10.3390/v16101621)
Supplement: Supplementary file 1 [file viruses-16-01621-s001.zip › viruses-3200994-supplementary.pdf]

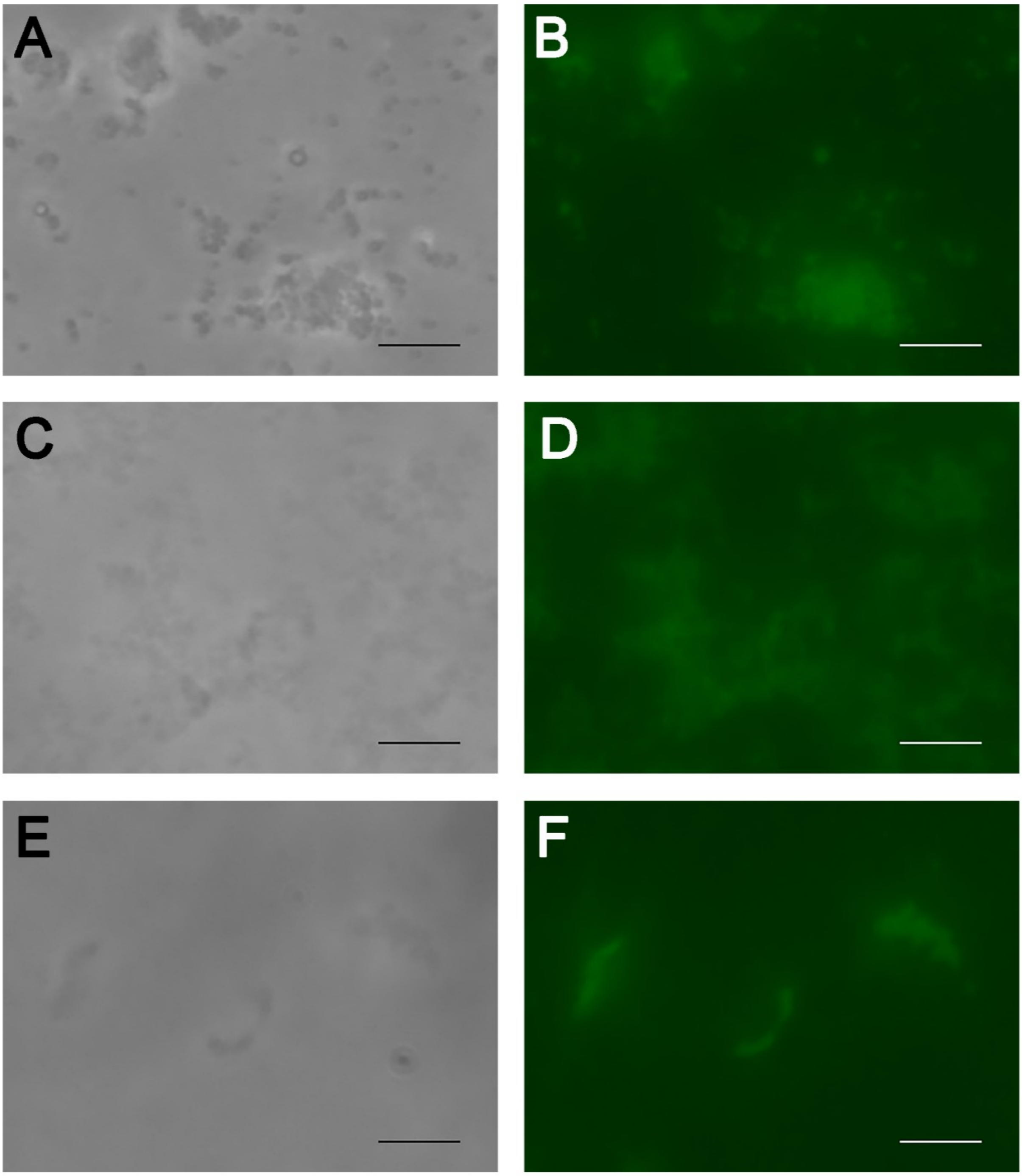

Figure S1. SP fluorescence labeling. A, B – TMV SP; C, D - AltMV SP$_{V}$; E, F – AltMV SP$_{VLP}$. A, C, E – phase contrast image; B, D, F – fluorescence image. Scale bar 5µm.

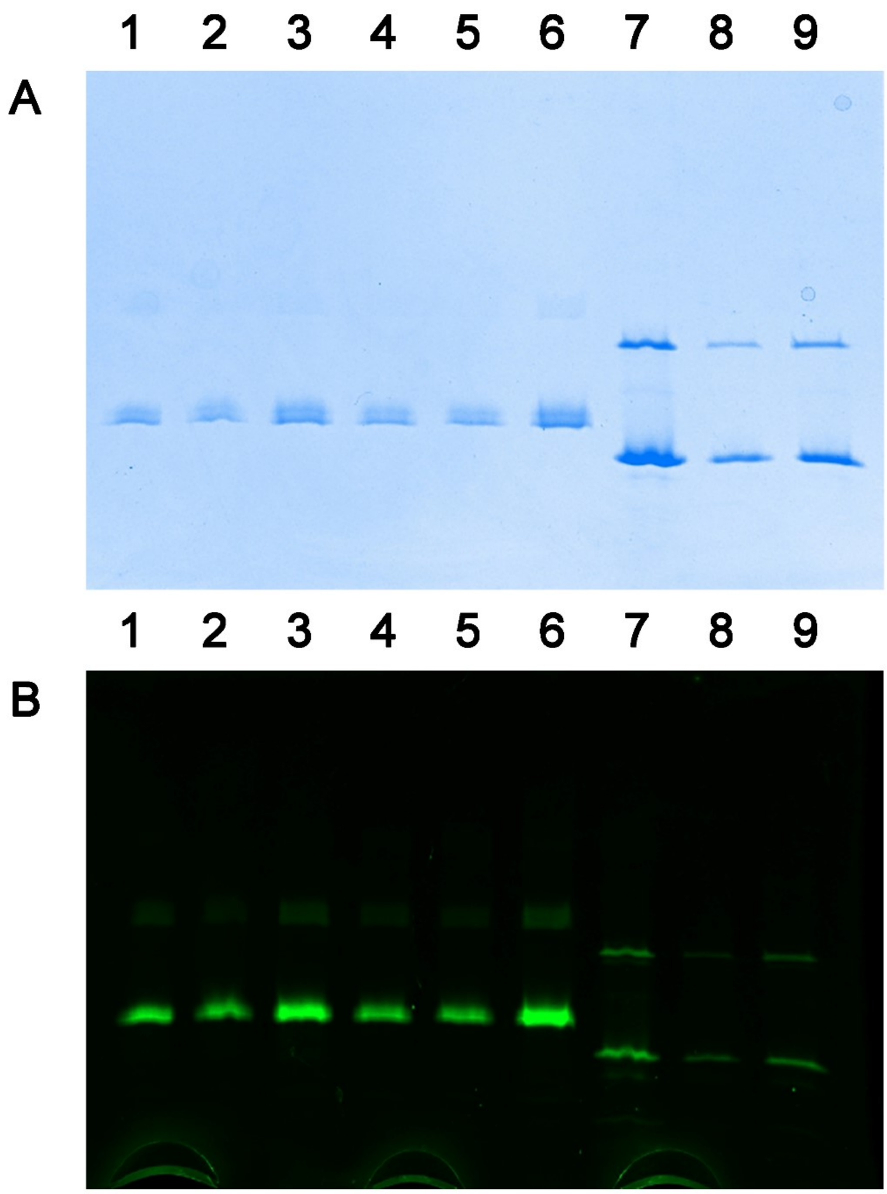

Figure S2. FITC-labeled coat protein within the following structures: lanes 1-3 – AltMV SP_{VLP}, lanes 4-6 – AltMV SP_{V}, lanes 7-9 – TMV SP. Lanes 1, 2, 4, 5, 8 9 – 1 μg, lanes 3, 6, 7 – 2 μg. A – staining with Coomassie G-250; B – visualization in UV-light.

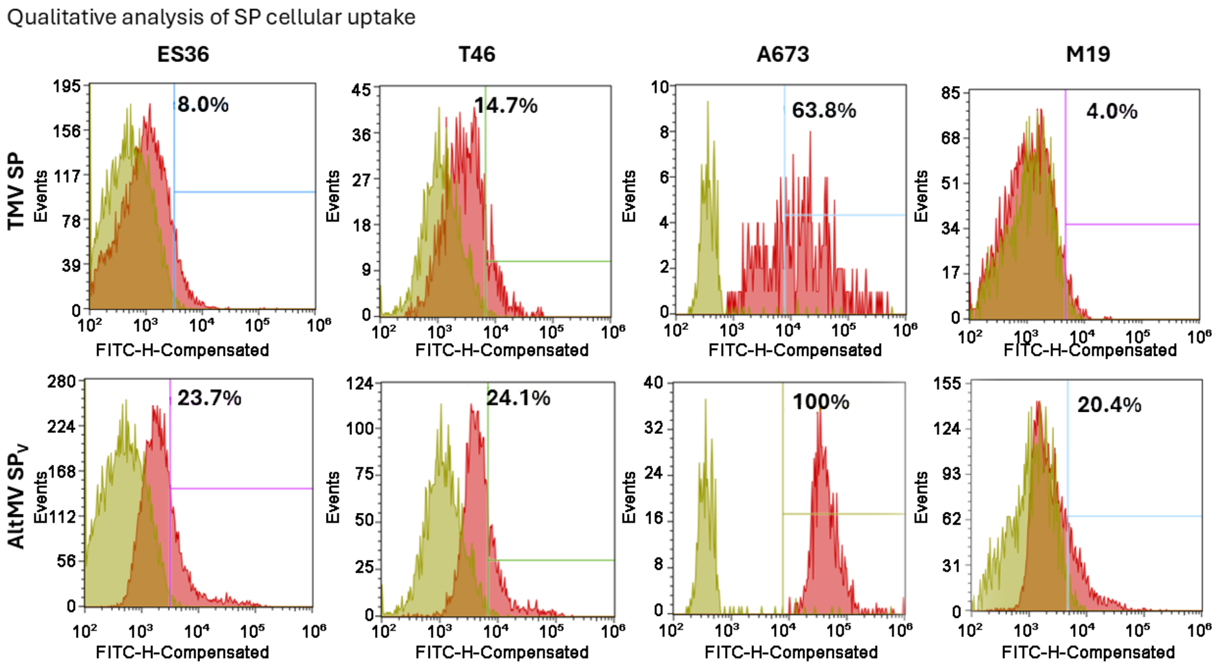

Figure S3. SP cellular uptake. Distribution of cells incubated with SP by fluorescence intensity for qualitative analysis. The events recorded by the device are plotted along the ordinate axis. The abscissa axis shows the fluorescence intensity detected in the channel. Control – cells without the addition of SP. The control is imposed on the results obtained during incubation with particles. It determines the boundary (vertical line), after which the result is considered positive. ES36, T46 – primary patient-derived Ewing sarcoma cells, A673 – established Ewing sarcoma line, M19 – normal fibroblast cell line.

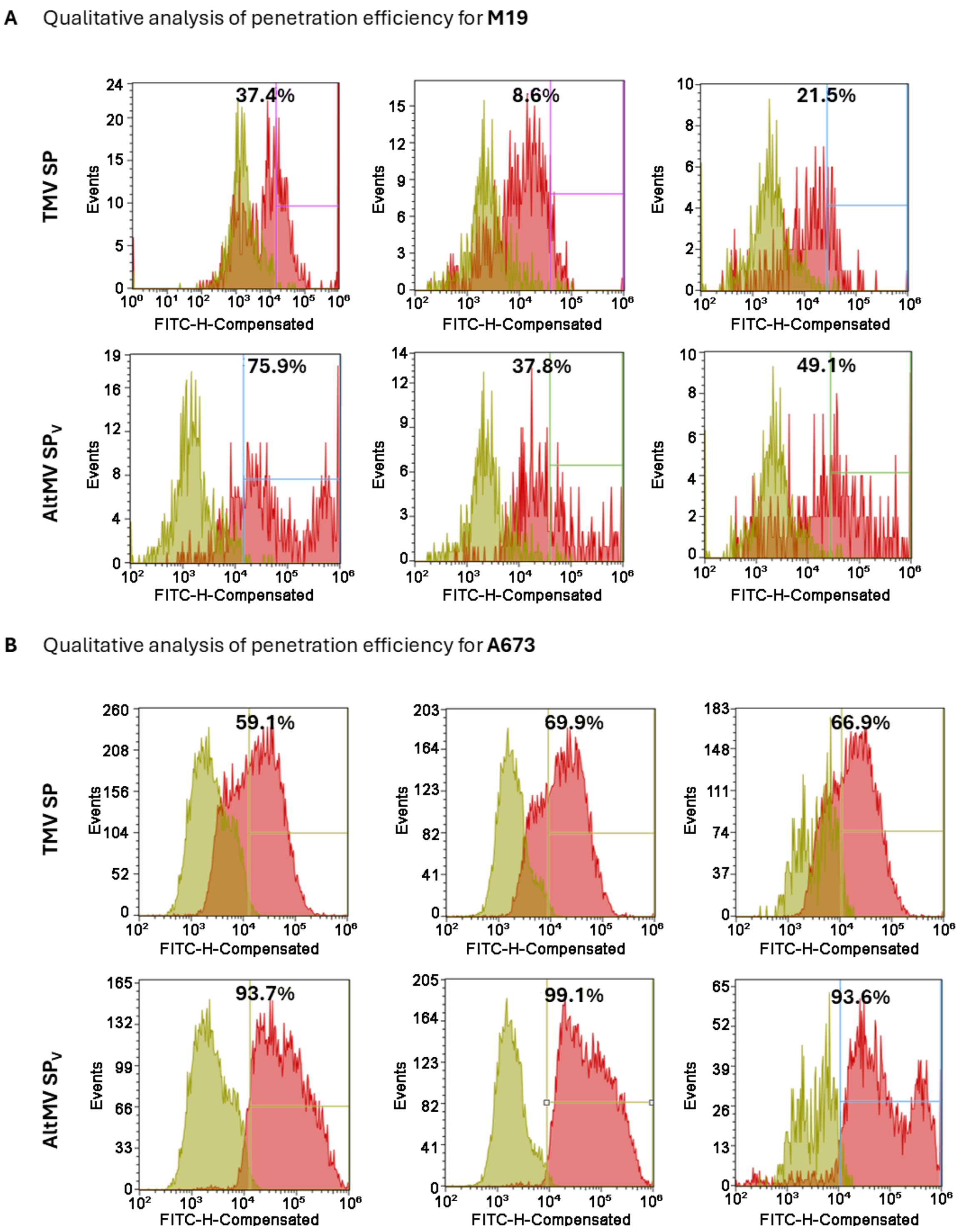

Figure S4. SP cellular uptake. Distribution of cells incubated with SP by fluorescence intensity for qualitative analysis. The events recorded by the device are plotted along the ordinate axis. The abscissa axis shows the fluorescence intensity detected in the channel. Control – cells without the addition of SP. The control is imposed on the results obtained during incubation with particles. It determines the boundary (vertical line), after which the result is considered positive. A) Distribution for SP added to M19 – normal fibroblast cell line B) Distribution of SP added to the A673 – established Ewing sarcoma line.

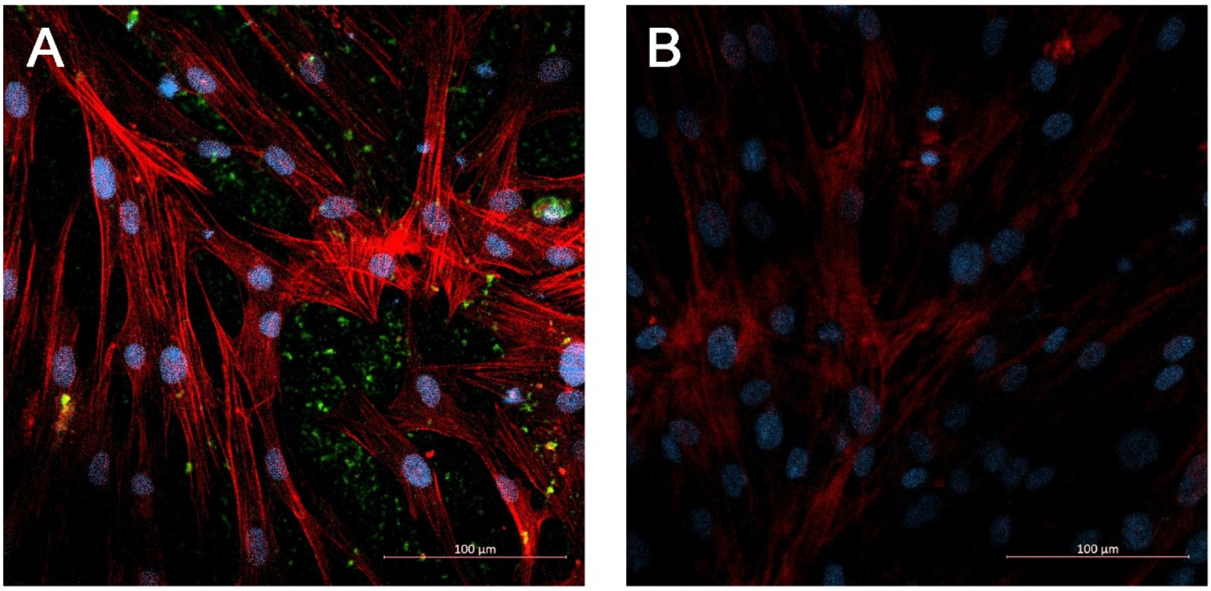

Figure S5. AltMV SPv retention inside primary Ewing sarcoma cells (ES36). A. AltMV SPv adsorption and penetration by ES36 cells. B. Negative control – ES36 without added AltMV SPv. Colocalization of the green and red signals produces a yellow signal, indicating the penetration of SP into the cell. The green signal demonstrates AltMV SPv that did not penetrate the cells and remained on the membrane and substrate surfaces. For the confocal microscopy, the magnification was 40.
